# Connaissances, attitudes et pratiques des populations de la ville de Lomé en matière de prévention de la bilharziose: cas du canton de Légbassito

**DOI:** 10.11604/pamj.2019.34.19.18918

**Published:** 2019-09-10

**Authors:** Kodjo Agbeko Djagadou, Toyi Tchamdja, Komi Dzidzonu Némi, Abago Balaka, Mohaman Awalou Djibril

**Affiliations:** 1 Service de Médecine Interne, Faculté des Sciences de la Santé, Université de Lomé, Lomé, Togo; 2 Service de Médecine Interne, Faculté des Sciences de la Santé, Université de Kara, Kara, Togo

**Keywords:** Endémie, connaissances, pratiques, bilharziose, Lomé, Endemic, knowledge, practice, schistosomiasis, Lomé

## Abstract

**Introduction:**

L'objectif de cette étude est de déterminer les connaissances, attitudes et pratiques des populations face à l'infestation bilharzienne.

**Méthodes:**

Il s'est agi d'une étude transversale descriptive et analytique sur les connaissances, attitudes et pratiques de la population du canton de Légbassito sur la bilharziose. L'échantillonnage calculé à la base de la population du canton par le logiciel DosBox 0.74 de Epi Info 3.5.4 a permis d'enquêter 380 personnes.

**Résultats:**

Ce travail a montré que sur 380 personnes enquêtés, 57,30% ne connaissaient pas les symptômes de la maladie, 40,10% ne connaissaient pas le mode de transmission de la maladie, 26,40% savaient qu'éviter tout contact avec les eaux de surface contaminées permet de prévenir la maladie, 18,20% fréquentaient les eaux douces de la localité dont 46,40% y allaient pour se baigner. Quant au mode d'évacuation des excréta, 90,80% évacuent les leurs dans des latrines, 1,30% urinent quelques fois dans les cours d'eau, 85,80% utilisent l'eau des forages pour les besoins domestique, 48,40% ne pensent pas pouvoir vivre avec un individu qui urine ou fait les selles avec du sang, 24,5% ne participaient pas souvent aux traitements de masse.

**Conclusion:**

Le canton de Légbassito est une zone endémique à l'infestation bilharzienne. La population adopte des attitudes et pratiques défavorables à l'élimination de la maladie telles les baignades aux heures chaudes, la miction dans les eaux douces et la fréquentation d'autres cours d'eau. Ces pratiques peuvent permettre de nouveaux cas d'infestation.

## Abstract (en)

**Introduction:**

The purpose of this study was to determine the knowledge, attitudes and practices of the populations relating to schistosomal infestation.

**Methods:**

We conducted a cross-sectional descriptive and analytical study of knowledges, attitudes and practices of the population of the township of Légbassito relating to schistosomiasis. The sampling calculated on the basis of the population of the township by the software DosBox 0.74 of Epi Info 3.5.4 allowed to investigate 380 subjects.

**Results:**

The study highlighted that out of 380 people investigated, 57,30% were not aware of the symptoms of the disease, 40,10% did not know the mode of transmission of the disease, 26.40% knew that avoiding any contact with contaminated surface water could prevent the disease, 18,20% came into contact with fresh waters of the area in which 46,40% of them went to bathe. Regarding sewage disposal, 90,80% used latrines, 1.30% urinated sometimes in the streams, 85,80% used wells water for domestic needs, 48.40% didn't think they could live with an individual whose urine or stool contains blood, 24.5% were not often involved in mass treatment.

**Conclusion:**

The township of Légbassito is an endemic area for schistosomiasis infestation. Population attitudes and practices are unfavorable to the elimination of the disease, such as bathing during the hot hours, urinating in fresh waters and coming into contact with other water courses. These practices could lead to new cases of infestation.

## Introduction

Maladies parasitaires très répandues dans le monde, surtout dans les zones tropicales, les bilharzioses ou schistosomoses constituent aujourd'hui la deuxième affection liée à l'eau, après le paludisme. La maladie se contracte le plus souvent à travers un contact entre l'homme et une eau douce (un ruisseau, un marigot, un fleuve, une rivière, un étang) contaminée par des cercaires. D'après l'Organisation Mondiale pour la Santé (OMS), la schistosomiase (ou bilharziose) est l'une des principales maladies transmissibles ayant des répercussions sanitaires et socio-économiques majeures dans plusieurs pays en voie de développement [1]. Malgré les efforts de lutte menés par la plupart des programmes des pays endémiques, notamment ceux d'Afrique sub-saharienne, on estime à 200 millions le nombre de personnes actuellement infectées dont une proportion importante est composée d'enfants de moins de 14 ans. Le nombre de personnes présentant les symptômes de la maladie est estimé à 120 millions dont 20 millions sont atteintes d'une forme grave et invalidante [2]. Au Togo, les études épidémiologiques révèlent la présence de deux espèces: 1) *Schistosoma haematobium* agent de la bilharziose génito-urinaire; *Schistosoma mansoni* agent de la bilharziose intestinale.

Les premières données sur la bilharziose remontent aux années cinquante. En 1960, cette endémie devient présente dans les 10 (dix) districts que comptait le Togo avec deux zones particulièrement sensibles d'une part les districts de Tsévié et Aného dans la région maritime, d'autres parts les districts de Lama-kara, Pagouda et Niamtougou dans la région de la Kara [3]. En 2009, une cartographie des Maladies Tropicales Négligées (MTN) dont la schistosomiase a été élaborée selon les directives de l'OMS, et un traitement de masse à la chimiothérapie préventive est organisé selon une fréquence recommandée. Le programme MTN fait ses interventions annuelles et biannuelles sur toute l'étendue du territoire en fonction des spécificités en rapport avec la prévalence de la bilharziose dans chaque district. Suite à l'élaboration de la cartographie des MTN, la schistosomiase a montré en 2009 une prévalence de 23% qui a nécessité un traitement de masse au praziquantel dans les régions centrale, de la kara et des savanes en 2010. Les autres régions (maritime et plateaux) n'ont connu le traitement qu'à partir de l'année 2011. Ce traitement effectué a permis d'obtenir une baisse importante de la prévalence jusqu'à 5% en 2015 dans les régions sanitaires centrale, de la kara, des plateaux et des maritimes. Dans la région sanitaire Lomé-Commune, le district du Golfe a enregistré une baisse de la prévalence de 5% en 2009 à 2,4% en 2015. Malgré cette baisse de la parasitose dans le district, seul le canton de Légbassito enregistre des chiffres croissants, passant de 0,07% en 2009 à 0,27% en 2015.

C'est face à ce constat que nous avons préféré orienter notre étude dans ce canton du district sanitaire du Golfe afin de déterminer les connaissances, attitudes et pratiques des populations face à l'infestation bilharzienne. Plus spécifiquement, il s'agissait: 1) identifier les facteurs de risque liés à la transmission de la maladie; 2) identifier les problèmes de santé publique posés par la bilharziose; 3) apprécier le niveau de participation de la population aux traitements de masse; 4) apprécier des mesures préventives et correctives pour baisser la morbidité de la maladie.

## Méthodes

Il s'est agi d'une étude transversale descriptive et analytique menée dans le canton d'Agoe-Légbassito qui abrite deux étangs d'eau. La période d'étude a été du 12 mars au 28 avril 2018. La population de Légbassito est estimée en 2018 selon les statistiques effectuées par la Direction Préfectorale de la Santé (DPS) du Golfe, est d'un effectif de 29909 habitants. L'échantillonnage a été déterminé à partir de l'effectif de la population du Canton de Légbassito en utilisant le logiciel DosBox 0.74 de Epi Info 3.5.4 avec une marge d'erreur de 5%. Ainsi le calcul effectué à un intervalle de confiance de 95% par ce logiciel, nous a permis de trouver un échantillon minimal de 379 personnes. La sélection dans chaque quartier a été réalisée par l'échantillonnage systématique dans les concessions à partir d'un point de départ déterminé au hasard et en tenant compte du pas de sondage calculé pour chaque quartier. Les entretiens ont été réalisés avec tous les ménages au sein d'une concession. La collecte des données a été effectuée par des entretiens individuels au niveau des ménages qui ont constitué l'échantillon. Il s'est donc agi d'une administration directe de la fiche d'entretien par un enquêteur formé après obtention du consentement des enquêtés. Les sujets âgés de moins de 8 ans et ainsi que ceux qui ne résidaient pas dans le canton étaient exclus de l'étude.

## Résultats

### Données sociodémographiques

Nous avions enquêté 380 personnes dont 203 de sexe féminin ce qui correspondait à un sex-ratio (H/F) de 0,87. L'âge moyen était de 34 ans avec des extrêmes de 7 et 60 ans. Par rapport au niveau d'instruction, les enquêtés ayant le niveau primaire étaient les plus représentés à 56,60% suivi du niveau secondaire 32,40%.

### Niveau de connaissances des enquêtés sur la bilharziose

L'hématurie et parfois la rectorragie étaient les principaux symptômes cités respectivement dans 91,30% (n = 347) et 52,10% (n = 198). En ce qui concerne le mode de transmission de la maladie, 139 enquêtés soit 40,10% avaient déclaré ne pas connaître le mode de transmission de la bilharziose contre 69 personnes soit 19,90% qui reconnaissaient le contact avec les eaux de surface contaminées comme le principal mode de transmission de la maladie. Les mesures de préventions citées par les enquêtés sont consignées dans le Tableau 1. Au plan thérapeutique, 310 personnes soit 89,30% ont déclaré que la maladie peut être soignée, 221 enquêtés soit 71,30% reconnaissaient l'efficacité du traitement médical conventionnel comme le meilleur contre la maladie. Dans notre étude, 117 enquêtés soit 33,70% avaient mentionné l'école primaire comme ayant été la principale source d'informations sur la bilharziose.

**Tableau 1:** Répartition des enquêtés selon la connaissance sur la prévention de la maladie

	Fréquence	Pourcentage (%)
Eviter tout contact avec les eaux de surface	81	26,4
Sensibilisation des populations	10	3,3
Utiliser les latrines	9	2,9
Traiter les réservoirs de la maladie	52	16,9
Porter les préservatifs	23	7,5
Eviter le contact avec le porteur de la maladie	12	3,9
Se laver régulièrement les mains	11	3,6
Ne sait pas	75	24,4
Total	307	100,0

### Attitudes et pratiques de la population face à la bilharziose

*Fréquentation des eaux douces de la localité:* sur les 380 personnes qui constituent notre échantillon, 69 enquêtés soit 18,20% attestent être des utilisateurs des eaux douces de la localité. Sur les 69 enquêtés qui ont l'habitude de fréquenter les eaux douces du canton, 32 personnes soit 46,40% y vont pour se baigner. Par contre 10 enquêtés soit 14,50% déclarent les fréquenter pour faire la lessive ou pour arroser les cultures de leurs champs. La fréquentation des eaux douces est associée à l'apparition des principaux signes de la bilharziose comme le montre le Tableau 2.

**Tableau 2:** lien entre la fréquentation des eaux douces et l’apparition du sang dans les urines et/ou les selles

Fréquentez- vous une eau douce du canton?	Urinez-vous une fois ou fait les selles avec du sang?		TOTAL
	Oui	Non	
Oui	23	46	69
Non	32	279	311
TOTAL	55	325	380

khi^2^ = 22,40 avec p= 0,0000034

*Mode d'évacuation des excréta:* dans notre étude, 345 personnes soit 90,80% de nos enquêtés évacuaient leurs excréta dans des latrines et le reste (35 enquêtés) le font hors des latrines. Plus de soixante-neuf pourcent (69,70%) de nos enquêtés urinaient dans les douches et 28,90% dans la nature. Par contre, certains enquêtés (1,30%) urinaient quelques fois dans les étangs d'eau.

*Sources d'approvisionnement en eau:* la majorité de nos enquêtés soit 85,80% (n = 326) utilisaient l'eau des forages et 1,30% (n = 5) utilisaient les eaux superficielles pour leurs besoins domestiques.

*Attitudes des enquêtés vis-à-vis d'un souffrant de bilharziose:* dans notre étude, 48,40% de nos enquêtés ont déclaré ne pas pouvoir vivre avec un individu qui urine ou fait les selles avec du sang.

*Participation aux traitements de masse:* seul 287 (75,50%) de nos enquêtés participaient souvent aux campagnes de traitement d'éradication organisées par les autorités du pays.

## Discussion

### Niveau de connaissances des enquêtés sur la bilharziose

Dans notre étude, 91,30% de nos enquêtés reconnaissaient la présence du sang dans les urines ou les selles comme étant symptomatique de la bilharziose; 57,30% avaient déclaré ne pas connaître les symptômes de la maladie, autre que la présence du sang dans les urines et les selles. Ould Ahmedou [4] dans son étude trouve que 72,9% de ses enquêtés reconnaissaient la présence de sang dans les urines ou les selles comme étant une maladie et 61,90% affirmaient que les douleurs à la miction étaient les symptômes les plus ressentis dans la forme urogénitale. Ceci montre que les populations méconnaissent les autres signes en l'occurrence le syndrome de Katayama lorsqu'il y a une notion de bain infestant en eau douce. Ce syndrome est caractérisé par: fièvre > 38°C, signes cutanés (réalisant la dermatite urticarienne fugace), douleurs (céphalées, myalgies, arthralgies), toux, parfois dyspnée asthmatiforme, douleurs abdominales et diarrhée [5].

En ce qui concerne le mode de transmission de la maladie, 19,90% reconnaissaient le contact avec les eaux de surface contaminées comme le principal mode de transmission de la maladie mais 26,40% des enquêtés savaient que pour prévenir la maladie, il faut éviter tout contact avec ces eaux. Ces résultats montrent un déficit en connaissances sur le mode de transmission et les moyens de prévention de la maladie. Nos enquêtés reconnaissaient qu'un traitement médical pouvait être une solution pour la maladie dans 71,30% des cas et servirait à briser la chaîne de contamination des eaux douces. Ces résultats sont contraires à ceux de Yao [6] dont la médecine traditionnelle a été le moyen de traitement efficace évoqué dans 66,15% des cas. En effet selon l'OMS, les victimes sont infectées dans le cadre d'activités agricoles, domestiques, professionnelles ou récréatives courantes, comportant des expositions à une eau contaminée. La lutte contre la schistosomiase se concentre sur la réduction du nombre de malades au moyen de traitements périodiques à grande échelle des populations par du praziquantel; une démarche plus globale, incluant l'apport d'eau potable et d'un assainissement approprié ainsi que la lutte contre les gastéropodes, devrait aussi faire régresser la transmission [5].

Quant aux sources d'informations sur la maladie, 33,70% avaient mentionné l'école comme étant le lieu où ils ont acquis des connaissances sur la maladie. Les résultats trouvés par Ould Ahmedou Mohamed [4] étaient presque similaires aux notre où l'école était également la principale source d'information dans 24,20% des cas. Ceci dénote que l'information du public sur la bilharziose était faible. Une large utilisation des masses médias ainsi que l'organisation des causeries dans les villages et les quartiers pourraient contribuer à la réduction de la transmission.

### Attitudes et pratiques face à la bilharziose

Les eaux de surface de notre zone d'étude étaient fréquentées par 18,20% de nos enquêtés et 46,40% de ces utilisateurs y vont pour se baigner. Aussi, le lien entre le sexe et la fréquentation des eaux douces du canton étant statistiquement significatif indique que le sexe masculin fréquente plus ces eaux pour la baignade et les activités champêtres. Ould Ahmedou Mohamed [4] ont trouvé des résultats similaires dont 46,10% des enquêtés allaient au fleuve pour la baignade. Ces résultats montrent que la baignade est une pratique servant d'amusement surtout aux enfants en âge scolaire d'une part et que les travaux champêtres occasionnent le contact avec les eaux douces d'autre part. La fréquentation des eaux douces du canton est un facteur qui expose les utilisateurs à la maladie.

En ce qui concerne le mode d'évacuation des excréta 09,20% de nos enquêtés font les selles en dehors des latrines par manque de latrine ou volontairement et 1,30% urinaient dans les eaux de surface. Yao [6] avait trouvé pratiquement un résultat similaire où 1,94% des enquêtés urinaient quelques fois dans les cours d'eau. Cette pratique peut être une source de contamination des eaux par des œufs de schistosomes si ces personnes sont déjà infestées. En cherchant la relation entre le lieu d'évacuation des excréta et la fréquentation des eaux douces de la localité, nous remarquons qu'il existe un lien statistiquement significatif entre ces deux variables. On peut donc conclure que faire des selles en dehors des latrines et uriner dans les eaux de surface, sont des pratiques qui concourent à la transmission de la maladie à tous ceux qui mettent pied dans ces eaux.

Pour s'approvisionner en eau, 1,30% utilisaient les eaux douces pour leurs besoins domestiques. Nos résultats sont strictement inférieurs à ceux de Yao [6] qui trouvait que 42,80% des enquêtés préféraient les eaux provenant des cours d'eau pour les besoins domestiques à cause de l'éloignement de la pompe a motricité humaine. L'usage des eaux douces pour les activités domestiques met ces personnes en contact avec lesdites eaux et lorsqu'elles sont infestées, elles favorisent la transmission de la maladie.

Pour éradiquer la maladie, des traitements de masse (TDM) au praziquantel sont périodiquement organisées par les autorités mais force est de constater que dans notre étude, 24,50% des enquêtés ne participaient pas souvent à ces TDM. Le même constat est fait par Dembele Ibrahima [7] dans son étude où 62,20% n'acceptaient pas les traitements médicaux en cas de bilharziose puisque selon eux, seul le traitement traditionnel est la solution efficace. Ces pratiques ne garantissent pas une rupture de la chaîne de transmission de la maladie ce qui pourrait entretenir l'endémicité bilharzienne dans le canton.

## Conclusion

Cette étude s'est proposé d'évaluer les connaissances, attitudes et pratiques des populations du canton de Légbassito qui est une zone endémique à l'infestation bilharzienne. Elle a permis de relever un déficit sur les connaissances des modes de transmission et de la prévention. La population adopte des attitudes et pratiques défavorables à l'élimination de la maladie telles les baignades et l'arrosage des champs avec les eaux des étangs et la miction dans les étangs. Ces pratiques peuvent permettre de nouveaux cas d'infestation.

### Etat des connaissances actuelles sur le sujet

- La bilharziose est un fléau qui date de la plus haute antiquité et constitue jusqu'à nos jours, un problème de santé publique. L'enquête réalisée par la Direction Générale de l'Action Sanitaire (DGAS) du Togo montre une augmentation de sa prévalence dans la préfecture d'Agoè où se trouve le canton de Légbassito.

### Contribution de notre étude à la connaissance

- Notre étude montre que la majorité des enquêtés (40,10%) et par ricochet une partie de la population ne connaissent pas le mode de transmission de la maladie et ne savent pas qu'éviter tout contact avec les eaux de surface contaminées permet de prévenir la maladie Il faut intensifier de façon compréhensible les campagnes de prévention.

## Conflits d’intérêts

Les auteurs ne déclarent aucun conflit d'intérêts.
